# Inflammation, Cancer and Immunity—Implication of TRPV1 Channel

**DOI:** 10.3389/fonc.2019.01087

**Published:** 2019-10-16

**Authors:** Joanna Katarzyna Bujak, Daria Kosmala, Iwona Monika Szopa, Kinga Majchrzak, Piotr Bednarczyk

**Affiliations:** ^1^Department of Physiological Sciences, Faculty of Veterinary Medicine, Warsaw University of Life Sciences, Warsaw, Poland; ^2^Department of Biophysics, Warsaw University of Life Sciences, Warsaw, Poland

**Keywords:** TRPV1, capsaicin, immune cells, LPS, capsazepine, ion channels

## Abstract

Process of inflammation and complex interactions between immune and cancer cells within tumor microenvironment are known to drive and shape the outcome of the neoplastic disease. Recent studies increasingly show that ion channels can be used as potential targets to modulate immune response and to treat inflammatory disorders and cancer. The action of both innate and adaptive immune cells is tightly regulated by ionic signals provided by a network of distinct ion channels. TRPV1 channel, known as a capsaicin receptor, was recently documented to be expressed on the cells of the immune system but also aberrantly expressed in the several tumor types. It is activated by heat, protons, proinflammatory cytokines, and associated with pain and inflammation. TRPV1 channel is not only involved in calcium signaling fundamental for many cellular processes but also takes part in cell-environment crosstalk influencing cell behavior. Furthermore, in several studies, activation of TRPV1 by capsaicin was associated with anti-cancer effects. Therefore, TRPV1 provides a potential link between the process of inflammation, cancer and immunity, and offers new treatment possibilities. Nevertheless, in many cases, results regarding TRPV1 are contradictory and need further refinement. In this review we present the summary of the data related to the role of TRPV1 channel in the process of inflammation, cancer and immunity, limitations of the studies, and directions for future research.

## Introduction

Cancer is the second cause of death in the world according to the World Health Organization. Recently, more attention is paid to the correlation between the process of inflammation and crosstalk between immune and tumor cells. Presence of specific immune cells that produce anti- or proinflammatory cytokines shapes the tumor microenvironment (TME) and have a great impact on tumor development outcome (1, 2). Therefore, current therapeutic approaches more often focus on targeting TME and harnessing the immune system to eradicate tumors. Consequently, the knowledge about specific molecules or their ligands that can hamper tumor growth, induce apoptosis, modulate immune response or influence tumor microenvironment, are of special interest for the medical field.

Ion channels are widely known to be involved in many biological processes in both excitatory and non-excitatory cells. They exhibit broad tissue distribution and their expression is not limited solely to the plasma membrane. Some of the ion channels are expressed in the inner compartments of the cells such as mitochondria where they play a fundamental role in the regulation of cell death and cytoprotection (3). Furthermore, increasing evidence indicates that ion channels have implications in cancer cell proliferation, survival and migration (4, 5). For this reason, ion channels emerged recently as potential pharmacological targets for the treatment of several disorders including neoplastic disease.

Transient Receptor Potential (TRP) ion channels are a family of non-selective cation channels that in vertebrates are represented by 28 different members divided into six subfamilies namely: Canonical (TRPC), Vanilloids (TRPV), Melastatins (TRPM), Mucolipins (TRPML), Polycystins (TRPP), and Ankyrin (TRPA1). They are receptors of multiple different stimuli such as temperature, pH, ROS, osmotic stress, and bacterial toxins. Furthermore, their activity is regulated by phytochemical compounds such as capsaicin, piperine, geraniol, or menthol (6). TRP ion channels were primarily associated with sensory nerve cells however recent findings showed that their expression is also present in other cell types including immune cells such as dendritic cells, macrophages, or T lymphocytes (7).

TRPV1 was the first identified member of the vanilloid receptor subfamily of TRP ion channels and to date is the most extensively studied. It was characterized in 1997 by Julius group as a receptor of capsaicin, a pungent compound from chili peppers (8). Further studies revealed that TRPV1 is also activated by temperature (with threshold ~43°C), protons and bacterial toxins such as lipopolysaccharide (LPS) (9, 10). For this reason, TRPV1 was associated with thermoception and nociception. Additionally, it is important to underline that several factors can influence activation threshold of TRPV1. For instance, in the presence of the divalent ions such as Mg^2+^ from the extracellular side, TRPV1 was shown to be activated at lower temperatures (11).

Expression in a wide range of tissues, such as skin, airways, gastrointestinal tract, urinary epithelial cells, pancreatic B cells and in the immune cells, underlines the important role of TRPV1. Currently it is known, that TRPV1 channel is not only involved in the thermal and pain sensation but also in other processes such as T cells activation, urinary bladder functions, insulin sensitivity, or airway hypersensitivity (12–19). TRPV1 was shown to be implicated in neurogenic inflammation, neuropathic pain, autoimmune disorders, cancer and immune cells functioning. To date, TRPV1 agonist, capsaicin or resiniferatoxin, and antagonists such as capsazepine (CPZ), BCTC, SB-705498, or NEO6860 were tested to treat migraine, osteoarthritis, overactive bladder, atopic dermatitis, and neuropathic pain (clinicaltrials.gov). Furthermore, in some studies capsaicin-induced TRPV1 activation was also associated with anti-inflammatory and anticancer effects. Thus, identification of novel modulators of TRPV1 activity might be beneficial to medical field.

TRPV1 channel is involved in the regulation of calcium signaling, crucial for many cellular processes including proliferation, apoptosis, secretion of cytokines or T cell activation. Furthermore, TRPV1 appears as a polymodal receptor that takes part in cell-environment crosstalk. Consequently, it can influence not only cell behavior but also cell fate and potentially can shape the antitumor response.

Therefore, TRPV1 might offer new therapeutic option in clinical applications against neoplastic diseases and pain treatment. Nevertheless, its involvement in the immune and cancer cells functioning is still under investigation. Herein, we present a review regarding the role of the TRPV1 channel in the process of inflammation, cancer, and immunity.

## Role of the TRPV1 Channel in the Process of Inflammation

Inflammation is a process characterized by pain, swelling, increased temperature, and redness that can be induced by pathogen infection or tissue damage. The main role of the inflammation is to stimulate the cells to fight against pathogens and regenerate destroyed tissue. It is tightly correlated with the action of immune cells and secretion of pro-inflammatory factors (cytokines, chemokines) (20). Currently, the process of inflammation is gaining more attention as it became linked to many pathological conditions including metabolic, neurogenerative, autoimmunological disorders but also tumorigenesis and progression of cancer (21–24).

TRPV1, as a pain and heat sensor expressed at high level in C-fibers associated with neurogenic pain, was primarily associated with neurogenic inflammation (25). However, expression of TRPV1 on both mRNA and protein level was also found on non-neural cell types and for this reason, recent studies address the role of vanilloid receptor in other inflammatory disorders such as asthma, rheumatoid arthritis or inflammatory bowel diseases (26–28).

The role of TRPV1 in the process of inflammation is vague. Primarily TRPV1 activation was associated with the induction of inflammatory response but recent studies demonstrated also its anti-inflammatory properties. TRPV1 was associated with the process of inflammation based on the studies that showed: (1) overexpression of V1 in inflamed tissues, (2) alleviation of inflammation by use of TRPV1 antagonist or genetic ablation or (3) correlation between TRPV1 activation and expression of proinflammatory cytokines.

### Association of TRPV1 Activation With Proinflammatory Responses

Increased expression of TRPV1 channel on both mRNA and protein level has been reported in several chronic inflammatory diseases for instance in the lung tissue of murine model of chronic asthma (29, 30), in synovial fibroblasts from patients with rheumatoid arthritis and osteoarthritis (31) or in esophageal mucosa in the course of non-erosive reflux disease (NERD) and erosive esophagitis (32, 33). Interestingly, in many studies inflammatory response was alleviated by administration of TRPV1 antagonists or TRPV1 siRNA which further underscore its role in the process. Nevertheless, further studies should also address the cell type in which TRPV1 is expressed in order to decode the true role of TRPV1 in the process of inflammation. Studies by Engler et al. (31) showed the increased expression of TRPV1 by qPCR and Western Blot analysis in the synovial fibroblasts isolated from patients with osteoarthritis and rheumatoid arthritis. Yet, in many studies the analysis was performed on entire organs (e.g., lung tissue) which contains a mixed cell population. Thus, the overall effect should be considered as a result of neuronal, immune and other cell types interactions.

In the studies by Ma et al. (34) exposure of human esophageal epithelial cells (HET-1A) to acidic pH (5.0) increased TRPV1 expression and led to ATP release and upregulation of inflammatory cytokines such as IL-8, Monocyte Chemoattractant Protein 1 (MCP-1), and Macrophages Inflammatory Protein-1α (MIP-1α). Silva et al. (33) showed that the development of esophageal inflammation could be blocked by TRPV1 antagonist SB366791 in the mouse model of NERD. These results suggest that TRPV1 is a potential pharmacological target in patients with NERD.

Also in mouse model of chronic asthma, administration of TRPV1 antagonist capsazepine (CPZ) or TRPV1 siRNA attenuated airway inflammation, hypersensitiveness, as well as reduced levels of cytokines IL-4, IL-5, IL-13 (type 2 T helper cytokines), and Thymic Stromal Lymphopoietin (TSLP), IL-25, IL-33 (epithelial cell-derived cytokines) (30). In urban particle matter and formaldehyde (FA) induced murine asthma model upregulated expression of TRPV1 was associated with enhanced release of pro-inflammatory neuropeptides such as substance P and Calcitonin Gene–Related Peptide (CGRP), which contributed to neurogenic inflammation. CPZ treatment, in turn, effectively reduced the level of pro-inflammatory neuropeptides (29).

Despite that in many studies CPZ was used as a TRPV1 antagonist, the results has to be treated with caution. CPZ recently was found to activate TRPA1 channel, which along with TRPV1 is involved in nociception and inflammation. Kistner et al. (35) found that CPZ administration led to attenuation of inflammation in the model of murine colitis. Nevertheless, authors demonstrated that the effect was TRPV1-independent and rather associated with desensitization of TRPA1. Furthermore, CPZ was also documented to block voltage-activated calcium channels (36). Thus, the use of CPZ to assess the role of TRPV1 in the process of inflammation might be speculative. To note, FA is known TRPA1 activator, thus the assessment of the TRPV1 role in the model of FA-induced inflammation should be done with caution. McNamara et al. (37) clearly demonstrated that formalin exerts its effect through TRPA1 not TRPV1 since the effect was abolished in TRPA1^−/−^ KO mice.

In the studies by Hoffmeister et al. (38) TRPV1 was linked with nociception and inflammation in the acute gout attacks. Injection of monosodium urate into rat joints induced hyperalgesia, allodynia, leukocyte infiltration and IL-1β production, which were inhibited by the co-administration of TRPV1 antagonist SB366791. Injection of TRPV1 antagonist also reduced nociception and associated pain-like behavior. Interestingly, monosodium urate crystals increased expression of TRPV1 in the articular tissue, which was correlated the peak of inflammatory response and nociception (38). Furthermore, deletion of TRPV1 gene was shown to attenuate synovial inflammation, bone erosion, cartilage damage in the course of rheumatoid arthritis (26).

In hairless mice with induced atopic dermatitis TRPV1 antagonist PAC-14028 administered as a cream improved skin barrier functions and restored expression of epidermal differentiation markers. Additionally, it significantly inhibited inflammation by decreasing the expression of IL-4 and IL-13 as well as expression of serum IgE (39).

A systemic inflammatory response in many cases is associated with a pathological condition known as sepsis. To date, the best models to study sepsis is cecal ligation and puncture (CLP) and LPS intravenous administration model. CLP is based on perforation of the cecum followed by microbial infection and subsequent severe immune response. TRPV1 was shown to be involved in the course of sepsis which was firstly proven by Bryant et al. (40) who suggested an important role of nociceptive system in response to infection. In the studies by Ninomiya et al. (41) pre-treatment of LPS-activated macrophages with TRPV1 antagonists AMG 9810 and CPZ reduced production of pro-inflammatory cytokines IL-6, IL-1β, and IL-18 and COX-2 expression. In mice, with LPS-induced lung injury, CPZ pre-treatment prevented the increase in respiratory system resistance, tissue damping, and decreased the area of collapsed lung parenchyma (42).

LPS was shown to activate TRPV1 but with lower potency than TRPA1. Furthermore, also other TRP ion channels (for instance TRPV4) were shown to be affected by LPS treatment (10). Thus, it has to be taken into consideration that the effects of LPS and TRPV1 pharmacological blockers might be the result of the interaction of several overlapping factors. The data on potential proinflammatory effect of TRPV1 activation or blockade is summarized in Table 1.

**Table 1:** Effects of TRPV1 activation (capsaicin) and blocking/genetic deletion on the process of inflammation.

	**Activator/blocker**	**Model**	**Dose**	**Time**	**Outcome**	**References**
Anti-inflammatory action	Capsaicin	Rats with induced sepsis	75 mg/kg	Injection on day 1	↓ rat's mortality	(40)
			50 mg/kg	Injection on day 2		
		Mice with LPS-induced bone inflammation	30 μM	24 h	↓ prostaglandin E production ↓ inflammation ↓ bone resorption	(43)
		Human umbilical vain endothelial cells (HUVEC) treated with LPS	3–10 μM	6 h	↓ cytokine/chemokine production ↓ adhesion molecule expression ↓ NF-κB activation ↑ NO production ↑ eNOS phosphorylation	(44)
	Capsazepine	Mice with LPS-induced lung injury	15 mg/kg	Single dose injection	↓ tissue damping during endotoxemia ↓ respiratory system resistance ↓ area of collapsed lung parenchyma	(42)
		LPS-activated murine macrophage-like cells (J774.1)	10 μM	Preincubated with CPZ 30 min before LPS	↓ pro-inflammatory cytokines production ↓ COX-2 expression	(41)
		Mice with surgically induced non-erosive reflux disease	5 mg/kg per injection	Injections daily for 7 days	↓ esophageal inflammation	(33)
		Formaldehyde and PM induced mice asthma model	3 mg/kg	Injections on day 1,7, and 14	↓ pro-inflammatory neuropeptides	(29)
		Rats with LPS-induced hypotension	3 mg/kg	Single dose injection 5, 10, or 25 min before LPS injection	↓ arterial blood pressure ↓ levels of substance P, norepinephrine, and epinephrine ↓ animals survival rate	(45)
		Mice with chronic asthma	50 μg	Injections daily for 3 months	↓ airway inflammation ↓ hypersensitiveness ↓ levels of cytokines	(30)
	TRPV1 siRNA		50 μg	administrated intranasally 2 times per week once per day		
	SB366791	Adult male Wistar rats	10 nmol/site	Single injection	↓ nociception ↓ hyperalgesia, ↓ allodynia, ↓ leukocyte infiltration	(38)
		Mice with surgically induced non-erosive reflux disease	3 mg/kg per injection	Injections daily for 7 days	↓ esophageal inflammation	(33)
	AMG9810	LPS-activated murine macrophage-like cells (J774.1)	10 μM	Preincubated 30 min before LPS administration	↓ pro-inflammatory cytokines production ↓ COX-2 expression	(41)
	PAC-14028	Hairless mice with induced atopic dermatitis	1.0% PAC-14028 cream	Applied on skin twice a day for 11 days	↑ skin barrier functions ↓ inflammation ↓ IL-4, IL-13, IgE production	(39)
	TRPV1 genetic deletion	TRPV1-deficient mice with arthritis	-	-	↓ synovial inflammation, bone erosion, cartilage damage	(26)
Proinflammatory action	Acidic pH (5.0)	Human esophageal epithelial cells (HET-1A)	-	12-min on seven occasions over 48 h	↑ IL-8, MCP-1, MIP-1α production	(34)
	FA	Formaldehyde (FA) and PM induced mice asthma model	2.44 ppm	for 3 h per day	↑ substance P, CGRP levels ↑ neurogenic inflammation	(29)
	PM		Exposure to PM <2.5 μm	8 h per day		
	Monosodium urate	Adult male Wistar rats	1.25 (mg/site) injected into the rat ankle joint	Single injection	↑ TRPV1 expression ↑ hyperalgesia, allodynia, leukocyte infiltration ↑ IL-1β production	(38)
	AMG-9810	Mice with LPS-induced sepsis	30 mg/kg per injection	Administrated 30 min before LPS injection	↑ sensitivity to LPS ↑ cardiac dysfunction	(46)
	TRPV1 genetic deletion	TRPV1-deficient mice with LPS-induced sepsis	-	-		
		TRPV1 KO Mice with allergic contact dermatitis	-	-	↑ TNF-α, IL-1β, and IL-6 production ↑ macrophages infiltration	(47)
		LPS-induced renal and hepatic inflammation in TRPV1 KO mice	-	-	↑ neutrophils infiltration ↑ TNF-α, IL-1β and IL-6 levels ↑ organ damage	(48)
		TRPV1KO mice with severe LPS-induced sepsis	-	-	↑ hypothermia, hypotension, organ dysfunction ↓ mononuclear cell integrity ↓ macrophage tachykinin NK(1)-dependent phagocytosis ↓ ROS levels ↓ bacteria clearance ↑ IL-6, IL-10, TNFα levels	(49)
		TRPV1 mice with LPS-induced peritoneal sepsis	-	-	↑ hypotension, hypothermia ↓ blood pressure ↑ liver edema	(50)

### Anti-inflammatory Role of TRPV1 Channel

Surprisingly, recent studies on the role of TRPV1 in the process of inflammation showed that pharmacological or genetic ablation of TRPV1 channel actually might aggravate the symptoms of inflammation.

Feng et al. (47) showed that lack of TRPV1 channel leads to cutaneous inflammation in the mouse model of allergic contact dermatitis. Authors demonstrated that TRPV1 deficiency was associated with upregulation of proinflammatory cytokines expression such as TNF-α, IL-1β, and IL-6 increased macrophages infiltration and simultaneous inflammation (47).

In rats injected with LPS, TRPV1 blockage with CPZ lowered arterial blood pressure, and levels of substance P, norepinephrine, and epinephrine, as a result lowering survival rate at 24 and 48 h after LPS administration (45). Nevertheless, in the light of more recent studies, such effect could be a result of CPZ influence on TRPA1 not TRPV1 (35).

**Figure 1:**
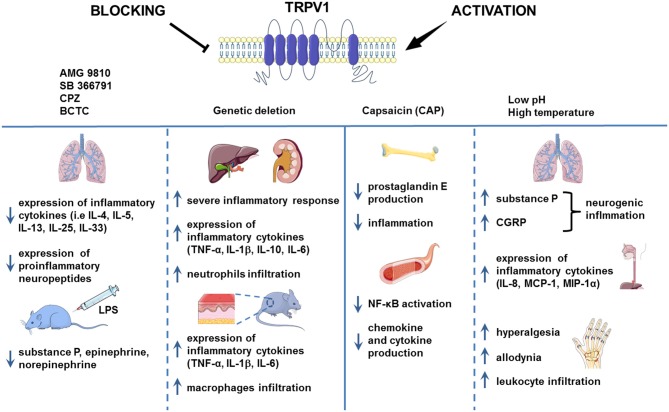
TRPV1 in inflammation. TRPV1 is widely known to be implicated in inflammation. However, the results of the studies regarding the role of TRPV1 channel in the process of inflammation are contradictory. In the chronic asthma model and mouse injected with LPS, the pharmacological blockade of TRPV1 decreased the level of proinflammatory cytokines. Genetic deletion of TRPV1, however, resulted in severe inflammation in the mouse model of LPS-induced renal and hepatic inflammation and allergic contact dermatitis. Activation of TRPV1 channel by H^+^ or formaldehyde was associated with aggravation of inflammation in the mouse model of asthma and human esophageal epithelial cells (HET-1A). Nevertheless, capsaicin administration led to the alleviation of inflammation symptoms as it was shown on the HUVEC cell culture and mouse LPS-induced bone inflammation model. Images adapted from Smart Servier Medical Art.

Also, in the model of LPS-induced renal and hepatic inflammation, TRPV1 KO mice exhibited significantly higher neutrophils infiltration, higher serum TNF-α, IL-1β, and IL-6 cytokines levels, more severe inflammatory response and subsequent exaggerated organ damage during endotoxic shock (48). This indicates that TRPV1 might actually have protective and anti-inflammatory role (Figure 1).

In the CLP sepsis model, TRPV1 knockout mice (TRPV1 KO) exhibited more severe LPS-induced sepsis compared with wild type. CLP TRPV1 KO mice showed significant hypothermia, hypotension, and organ dysfunction caused by decreased mononuclear cell integrity associated with apoptosis, macrophage tachykinin NK(1)-dependent phagocytosis, level of reactive oxygen species and bacteria clearance but increased IL-6, IL-10, and TNFα levels (49). Also, Clark et al. (50) showed that genetic deletion of TRPV1 in the murine model of LPS-induced peritoneal sepsis led to enhanced hypotension, hypothermia, decreased blood pressure and liver edema, thus, indicating that TRPV1 is involved in response regulation to sepsis and has an overall protective role.

Also, administration of TRPV1 agonists was shown to reduce inflammation. Bryant et al. (40) showed that injection of capsaicin reduced rat's mortality in the model of abdominal sepsis. Mice with LPS-induced bone inflammation treated with capsaicin exhibited suppressed prostaglandin E production, inflammation and subsequent bone resorption (43). Presence of capsaicin in HUVEC cells culture before LPS stimulation lowered cytokine and chemokine production, adhesion molecule expression, and NF-κB activation but increased nitric oxide (NO) production and endothelial nitric oxide synthase (eNOS) phosphorylation. Authors suggested that TRPV1 activation in endothelial cells is associated with activation of eNOS/NO pathway and consequent suppression of inflammation (44). Moreover, endogenous activation of cardiac TRPV1 during sepsis and endotoxemia led to the release of CGRP minimizing cardiac dysfunction (46). Summarizing, anti-inflammatory action of TRPV1 activation or blockade is presented in Table 1.

TRPV1 is widely known to have pro-inflammatory role, but growing evidence points to its anti-inflammatory and protective role as well. It is important to underline that involvement of TRPV1 in the process of inflammation is regulated at several levels starting from gene expression to post-transcriptional modifications and cellular compartmentalization. The process is also associated with many regulatory proteins and multiple inflammatory mediators, which might obscure the true role of TRPV1 in the process. Furthermore, TRPV1 can be expressed on different cell subsets such as primary sensory nerves or immune cells and the interaction between them may shape the final outcome of TRPV1 activation.

## TRPV1 and Implication in Cancer

### TRPV1 and Cancer Development

It is widely known that chronic inflammation is associated with tumorigenesis and aberrant calcium signaling promotes proliferation, metastasis, and cancer cell survival. TRPV1 is linked with both the process of inflammation and calcium signaling, thus, its contribution to cancer progression gained more attention.

Functional expression of TRPV1 was demonstrated in several tumor types including human breast cancer cell lines (MCF-7 and BT-20), human papillary thyroid carcinoma BCPAP cells, prostate cancer (LNCaP and PC-3), urothelial cancer cells, and glioma (51–55).

Weber et al. (54) showed that TRPV1 expression was upregulated in the several native breast cancers comparing to healthy tissue. On the contrary, downregulation of TRPV1 expression was associated with progression of urothelial cancer and thus potentially could be used as a prognostic marker (56). Also, studies by Vinuesa et al. (57) showed that TRPV1-deficient mouse more often developed colitis-associated cancer in the distal colon. It is surprising, especially that TRPV1 was suspected to be involved in inflammatory bowel diseases that actually predispose to colon cancer development. However, the exact role of TRPV1 in tumorigenesis is not clear. TRPV1 is sensitized by inflammatory mediators and its activation can lead to the release of calcitonin gene-related peptide (CGRP) and substance P, known proinflammatory factors. On the other hand, activation of TRPV1 might also lead to secretion of neuropeptides with anti-inflammatory properties such as somatostatin (58).

Although it was suggested that TRPV1 antagonism might be associated with a risk of skin tumor development via EGFR/Akt signaling, more recent study have shown that two commercially available TRPV1 antagonists (AMG-9810 and SB-705498) do not affect human keratinocytes proliferation *in vitro* as well as do not have an impact on the skin carcinogenesis in mouse study (59, 60). This confirms the possibility of using TRPV1 antagonists in the treatment of skin conditions such as pruritus associated with atopic dermatitis. The study of Hwang et al. (61) also demonstrated that capsaicin acts as a co-carcinogen in 12-O-tetradecanoylphorbol-13-acetate (TPA)-promoted skin carcinogenesis through the activation of EGFR/Akt signaling but without the involvement of TRPV1 channel.

### TRPV1 and Cancer Therapy

**Figure 2:**
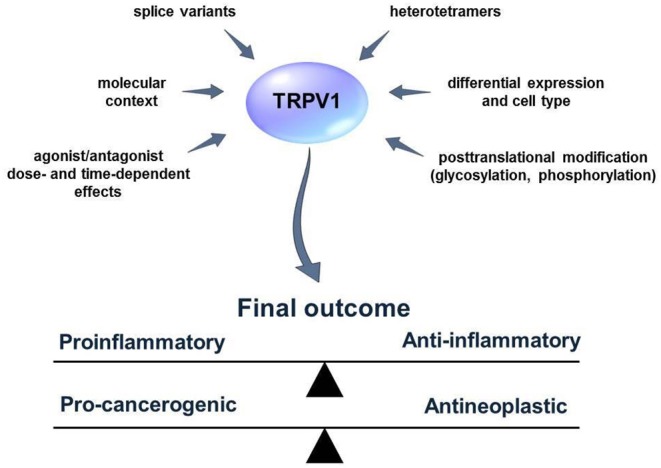
External and internal factors that influence TRPV1 activity and functions. Many aspects of the TRPV1 mode of action are not fully known. Several factors such as the existence of TRPV1 splice variants or heterotetramer formation can obscure the results of the studies concerning the role of TRPV1 in the process of inflammation and cancer. TRPV1 activators/blockers can exhibit synergistic/antagonistic or off-target effects with other elements of the living cell. Furthermore, TRPV1 might influence distinct cellular pathways and lead to cell-context depending effects. Defining those factors might help to predict the outcome of TRPV1 activation or blockage more accurately.

Several studies addressed the TRPV1 activation in anti-cancer therapy via harnessing the Ca^2+^ signaling. Activation of TRPV1 by capsaicin (150 μM), was shown to significantly reduce proliferation and induce apoptosis of aggressive triple-negative breast cancer cell line (SUM149PT) (54). On the other hand, studies by Pecze et al. (62) showed that despite the presence of the TRPV1 protein expression in the breast (MCF7, MDA-MB-231, BT-474) and prostate carcinoma (PC-3, Du 145, LNCaP) cell lines, capsaicin administration at 50 μM dose did not exhibit any cytotoxic effect. However, when cells were transiently transfected with cDNA coding human TRPV1, administration of lower doses of capsaicin (2 μM) caused significant Ca^2+^ accumulation in mitochondria, leading to apoptosis. Such discrepancies might result partially from complexity of cellular signaling pathways mediated and integrated by TRPV1 but also from other factors that influence TRPV1 functioning (Figure 2). Furthermore, capsaicin itself might act not only through TRPV1-dependent calcium signaling pathway. For instance, Hawng et al. (61) showed that the co-carcinogenic action of capsaicin in TPA-promoted skin carcinogenesis is associated with the activation of the EGFR/Akt pathway not Ca^2+^ signaling via TRPV1. Similarly, Bao et al. (63) demonstrated that in the osteosarcoma MG63 cells capsaicin treatment increases phosphorylation of AMPK and p53 in the TRPV1-independent manner. Also, Pramanik et al. (64) found that capsaicin inhibited β-catenin/TCF-1 signaling in the pancreatic cancer cells.

Also, little is known about functional expression of TRPV1 in cancer cells. To date, four splice variants of TRPV1 were described in the literature (65). The functional expression of TRPV1 splice variants in different cell types e.g., cancer or immune cells has to be addressed in future studies since it might have a great impact on the results. Pecze et al. (66), for instance, found expression of TRPV1 on both mRNA and protein levels in the human keratinocytes. However, they also show calcium overload associated cytotoxicity and subsequent keratinocytes death after CAP and RTX treatment at very high doses in a micromolar range (from 16 up to 300 μM) in contrast to nerve cells (nanomolar range). Interestingly, the authors found high mRNA expression of TRPV1b variant in keratinocytes but they were not able to show protein expression of both TRPV and TRPV1b. It might indicate no functional expression of the channel in the keratinocytes, however, it was not clearly stated whether the antibodies used in the study were able to recognize both splice variants of TRPV1. Furthermore, TRPV1b was documented to have a negative effect on the TRPV1 activation by capsaicin, heat, and pH as it was shown by Vos et al. (67) and thus in the cells with higher expression of TRPV1b over TRPV1, the effect of capsaicin treatment might not be visible when the low dose is applied. It is also speculated that different splice variants might undergo tetramerization, which is necessary for proper functioning of the TRPV1 ion channel. Also, it is suggested that TRPV1 forms heterotetramers with other TRP ion channels, for instance TRPA1 (68). This may produce unique properties in terms of activation and permeability of such heterotetramers. The effects of such a hetero-tetrameric assembly and its consequences in the progression of neoplastic diseases are yet to be determined.

Similarly, not only tetrameric assembly but also co-expression with other ion channels might have significant physiological effects. Weng et al. (69) demonstrated that regulation of association between TRPA1 and TRPV1 by transmembrane protein Tmem100 has a profound effect on pain perception. The authors found that close association between TRPA1 and TRPV1 leads to TRPV1-dependent inhibition of TRPA1 while in the presence of Tmem TRPA1-V1 association was weaker. Such results indicate that TRPV1 action in many cases cannot be considered separately from TRPA1.

Several studies have demonstrated that administration of chemotherapy along with TRPV1 activator—capsaicin, results in synergistic effect leading to increased apoptosis and suppression of tumor cell migration. Studies by Deveci et al. (70) showed that activation of TRPV1 is associated with significantly higher level of apoptosis in the MCF-7 human breast cancer cell line than in the cells treated with anticancer drug 5-Fluorouracil alone. Similarly, Nur et al. (71) revealed that the apoptotic effect of cisplatin was improved by activation of TRPV1 in the MCF-7 cell line. Furthermore, alteration of TRPV1 activity by capsaicin was shown to significantly reduce the migration and invasion of human papillary thyroid carcinoma BCPAP cells (55).

The mechanism by which activation of TRPV1 channel improves chemotherapy outcomes is yet to be determined. It is speculated that activation of TRP channels and its subsequent opening allows for direct permeation of small cytotoxic drugs through the pore (72). It is in accordance with studies by Hofmann et al. (73) who demonstrated that TRPV1 is involved in the uptake of anandamide, the endogenous cannabinoid and agonist of TRPV1 implicated in tumor-angiogenesis, by human endothelial colony-forming cells (ECFCs), and a human endothelial-vein cell line (E.A.hy926). The authors suggest that TRPV1 might create a pore that allows for passage of small molecules. Ortega-Guerrero et al. (74) performed a simulation which shows a high probability of doxorubicin permeation through TRPV1 but only when the channel is dilated. Based on these results, the authors implied that the TRPV1 channel might serve as a pathway for cancer therapy drug delivery systems. Despite these promising results, we cannot exclude that there is other, unknown mechanism involved in the transport of chemotherapeutic agents and small molecules or cell sensitization.

In many studies, capsaicin was used as a potent TRPV1 activator. However, it is important to distinguish between the effect of TRPV1 activation and the effect of the capsaicin itself. Studies by Gonzalez et al. (75) revealed that capsaicin treatment decreased the viability of the oral squamous cell carcinoma (OSCC) cells but the mechanism was independent of TRPV1 activation. Using calcium imaging, authors proved that TRPV1 channels were not functional in the OSCC cells and the cytotoxic effect of capsaicin and CPZ, was due to the promotion of reactive oxygen species (ROS) production by vanilloids. Similarly, Zhang et al. (76) showed that administration of capsaicin along with sorafenib leads to significant inhibition of the hepatocellular carcinoma (HCC) cells proliferation. The authors revealed, however, that the expression of TRPV1 was not detected in the HCC cells and sensitivity to sorafenib was increased due to capsaicin-dependent phosphorylation of ERK. Also, Kim et al. (77) showed that longer exposure to high doses of capsaicin (100 μM, 24 h) indeed is able to induce apoptosis in the gastric cancer cells (AGS, MKN45, and Hs746T). However, in the same time, lower doses of capsaicin (~20 μM, ~3 h) caused significant impairment of NK cells performance including the reduction in cytotoxic activity and cytokine production important for augmenting NK cell-mediated antitumor reaction. Most importantly, capsaicin-mediated dysfunction of NK cell was mostly TRPV1-independent (77). Bao et al. (63) demonstrated that activation of TRPV1 by capsaicin was essential to induce cytotoxic effects on osteosarcoma MG63 cells. Nevertheless, authors also showed that capsaicin treatment induces activation of the AMPK-p53 pathway which promotes apoptosis. Importantly, blocking TRPV1 activation with CPZ did not inhibit the phosphorylation of AMPK and p53, suggesting a TRPV1-independent action of capsaicin. Importantly, several studies documented inhibition of transient potassium currents but also store-operated calcium entry (SOCE) caused by capsaicin itself, especially at high concentrations (78, 79). Furthermore, capsaicin was shown to halt the cell cycle at G_2_/M phase and disrupt mitochondrial membrane potential leading to mitochondria-mediated apoptosis (80). Thus, capsaicin may exert cytotoxic effects without TRPV1 channel involvement.

On the contrary to the antitumor effect of capsaicin, Caprodossi et al. (81) have demonstrated that capsaicin administration upregulated genes associated with angiogenesis, invasiveness and metastasis processes in the human urothelial cancer cell line (5,637) that was TRPV1-deficient. The same cell line transfected with TRPV1 and treated with capsaicin showed significant increase in intracellular Ca^2+^ levels and subsequent growth inhibition and the increase of apoptosis (81). The effects of capsaicin and TRPV1 blockers on cancer cells are summarized in Table 2.

**Table 2:** Effects of the capsaicin and TRPV1 blockade on the cancer cells.

**Activator/blocker**	**Dose**	**Time**	**Outcome**	**Effect**	**References**
Capsaicin	20 μM	12–72 h	↑ apoptosis in human osteosarcoma MG63 cells ↑ CAP-induced mitochondrial dysfunction ↑ AMPK-p53 pathway activation but in TRPV1-independent mechanism	Anti-cancer activity	(63)
	100 μM	24 h	↑ intracellular Ca2^+^ levels ↑ apoptosis ↓ growth of human urothelial cancer cell line (5,637) transfected with TRPV1	Anti-cancer activity	(81)
	1 μM	10 min	↑ apoptotic effect of 5-FU in human breast cancer cells (MCF-7)	Anti-cancer activity	(70)
	100 μM	24 h	↑ apoptosis in the gastric cancer cell lines (AGS, MKN45, and Hs746T)	Anti-cancer activity	(77)
	20 μM	3 h	↓ NK cells performance, ↓ cytotoxic activity ↓ cytokine production TRPV1-independent pathway	Anti-inflammatory activity	(77)
	1 μM	2 min	↑ cisplatin induced apoptosis in human breast cancer cells (MCF-7)	Anti-cancer activity	(71)
	50 μM	24 h	- no cytotoxic effect in breast and prostate cancer cell lines	No activity observed	(62)
	2 μM	10 min	Breast and prostate cancer cell lines transfected with cDNA coding human TRPV1 ↑ Ca^2+^ accumulation in mitochondria ↑ apoptosis ↑ Na^+^–and Ca^2+^–dependent membrane disorganization	Anti-cancer activity	(62)
	150 μM	48 h	↓ proliferation ↑ apoptosis in breast cancer cell line (SUM149PT)	Anti-cancer activity	(54)
	25–100 μM	24 h	↓ migration and invasion of human papillary thyroid carcinoma cells (BCPAP)	Anti-cancer activity	(55)
	10 μM applied topically	Single dose	↑ induction of skin tumors in the model of TPA–promoted skin carcinogenesis (WT mouse and TRPV1KO mouse) TRPV1-independent mechanism	Procarcinogenic activity	(61)
TRPV1 genetic ablation	-	-	↑ number of tumors in the distal colon	Procarcinogenic activity	(57)
AMG-9810	Topical application of 1mg	Single dose	↑ mouse skin tumorigenesis ↑ EGFR expression ↑ Akt/mTOR signaling pathway ↑ increased proliferation in human keratinocytes N/TERT1 cell line	Procarcinogenic activity	(59)
	0.25–5 μM in the cell medium	24 h, 48 h, 72 h			
PAC-14028 SB-705498 AMG-9810	1–10 μM in the cell culture medium	24, 48, 72 h incubation	No EGFR/Akt/mTOR signaling pathway upregulation No effect on cell proliferation No impact on the skin carcinogenesis HEKn and HaCaT cells, ^*^in vivo studies on mouse model	No procarcinogenic activity	(60)
	1 mg in acetone applied on the gauze patch or applied directly on the skin	3 h			

The scientific data underlines the role of TRPV1 as a potential calcium signaling modulator and drug target against cancer.

## TRPV1 and Immunity

TRPV1 was found recently to be expressed in the cells of both innate and adaptive immune system including macrophages, dendritic cells, T lymphocytes, NK cells (49, 77, 82, 83). TRPV1 appeared to be a putative modulator of the immune cell functioning since it is involved in both calcium signaling and transduction of external stimuli such as temperature or pH. However, the data about its exact role in the immune system is not well-determined and, in many cases, the results are contradictory. Furthermore, based on the studies by Cavanaugh et al. (84, 85) that incorporated two lines of knock-in mice expressing reporter genes under endogenous TRPV1 promotor (TRPV1_Cre_ and TRPV1_PLAP−nlacZ_), TRPV1 mRNA expression was shown to be confined mostly to peptidergic sensory neurons but functional expression was also found in arteriolar smooth muscle cells. The results, therefore, do not support the hypothesis of TRPV1 expression in other cell types including immune cells. On the other hand, Xue et al. (86) drew attention to the fact that TRPV1 transcription might be regulated by a dual promotor system. Authors found that despite one of the promotors (P1) exhibit strong activity in sensory neurons, it was also active in the fibroblast cell line (3T3) in a lower but significant levels. For this reason, the authors did not rule out the possibility of TRPV1 expression in other cell types including PBMCs.

Calcium ions are well-known second messenger which in immune cell play a crucial role in activation, differentiation, proliferation, cytokine secretion, and effector functions (87, 88). Also, temperature and cationic milieu (including H^+^ concentration) were demonstrated to have a great impact on immune cells performance (89, 90). For instance, the elevated temperature was shown to enhance differentiation of T cell into effector cells while low pH is associated with impairment of T cell action (91, 92). Also, magnesium ions play important role in immunity and disruption of Mg^2+^ homeostasis was associated with immune system malfunctioning e.g., T cell-related immunodeficiency (93). Interestingly, studies by Cao et al. (94) showed that the presence of divalent cations such as magnesium alters TRPV1 activation threshold. Thus, TRPV1 seems to be a good candidate for immune cells modulation.

TRPV1 was reported to be involved in macrophages-associated defense mechanism. It was shown that treatment with capsazepine, an antagonist of TRPV1, significantly decreased the production of proinflammatory cytokines such as IL-6, IL-1β, and IL-18 and cyclooxygenase 2 (COX-2) in the LPS-stimulated murine macrophages (41). The data indicate that TRPV1 is involved in macrophages activity and that potentially TRPV1 antagonist can be used to suppress inflammatory responses and to treat sepsis. Also, TRPV1 activation by evodiamine (0.5 μM) or capsaicin (10 μM) in primary murine macrophages was linked with decrease in production of pro-inflammatory cytokines such as MCP-1 (Monocyte Chemoattractant Protein 1) and IL-6 induced by TNF-α while the treatment with TRPV1 antagonist, CPZ, abolish the anti-inflammatory effect (95). Deletion of TRPV1 in murine model caused impairment of proper macrophages-associated defense mechanism leading to systemic inflammation. Nevius et al. (96) showed that macrophages in the pancreatic lymph nodes (PLN) respond to orally administrated capsaicin and upregulate expression of anti-inflammatory cytokines such as IL-10. Interestingly, enhancement of macrophages population by capsaicin was linked with decrease in proliferation of autoreactive T cells. The authors demonstrated also that capsaicin did not inhibit proliferation of T cells from PLN in the TRPV1 KO mice which suggest that TRPV1 might be used as a pharmacological target against autoimmune diabetes (96).

Dendritic cells (DCs), similarly to macrophages, are cells of the innate immune system responsible for antigen presentation, stimulation of T cells and shaping the adaptive immune system. TRPV1 was found to be expressed on the murine dendritic cells and the activation by capsaicin was shown to stimulate DC, their maturation and migration to the lymph nodes of the wild type mice. Dendritic cells derived from the bone marrow of TRPV1^−/−^ mice did not undergo maturation after agonist treatment which indicated the pivotal role of TRPV1 in that process (97). On the other hand, studies by O'Connell et al. (98) have not confirmed the TRPV1 expression in the DCs. Authors were not able to elicit calcium influx on Ca^2+^-imaging experiment or change in membrane currents using electrophysiological techniques in DC treated with 1 μM capsaicin. The effect of capsaicin treatment was, however, pronounced in the sensory neurons and HEK293 cells expressing TRPV1. Also, the authors were not able to detect TRPV1 transcript in the DCs and thus they concluded that murine DCs do not express capsaicin receptor (98). It is worth to notice, that in the study by Basu and Srivastava (97) there was a dose-dependent effect of capsaicin on DCs maturation from WT mice with doses <10 μM having no significant effect. In the experiments performed by O'Connell et al. (98) 1 μM capsaicin was used. Discrepancies might arise from capsaicin doses, capsaicin treatment time or differences in the cell type (DC, neurons, transfected HEK cells). In DCs the expression of TRPV1 could be lower or they can express different splice variants of TRPV1 and thus they need a stronger signal to become fully activated. Also, we cannot exclude the TRPV1-independent effect of capsaicin on DCs, especially during prolonged exposures. Recent studies by Assas et al. (83) revealed the functional expression of TRPV1 in the murine splenic dendritic cells, macrophages and lymphocytes. Authors also proved that splenic DCs respond in a dose-dependent manner (12, 50, and 100 μM) to capsaicin by increase of calcium levels and subsequent release of CGRP (Calcitonin-Gene Related Peptide) and most importantly the reaction was TRPV1-dependent since the same effects were not observed in the cells isolated from the TRPV1^−/−^ mice. TRPV1 activation in DCs might, therefore, be important for maintaining immune homeostasis.

Despite that, gene expression of TRPV1 was found in neutrophils, capsaicin and low pH did not alter calcium influx. It can indicate no functional expression of TRPV1 channel in neutrophils (99). On the contrary, Schepetkin et al. (100) however showed that phytochemicals from *Ferula akitschkensis* modulate responses of human neutrophils partially via TRPV1. Thus, the authors demonstrated that TRPV1 might be functionally active in human neutrophils. Furthermore, Kose and Nazıroǧlu (101) showed the evidence of calcium fluxes in neutrophils in response to capsaicin that were decreased by both administration of N-acetyl cysteine or TRPV1 blocker capsazepine. The authors concluded that TRPV1 in neutrophils from patients suffering from polycystic ovary syndrome (PCOS) takes part in calcium entry and might be associated with calcium-entry dependent activation of neutrophils and release of pro-inflammatory cytokines. Nevertheless, the knowledge about TRPV1 in neutrophils is very limited and thus it requires further refinement.

Most of the cells from the lymphoid lineage, namely T cells, B-cell and NK cells, express TRPV1 channel. Kim et al. (77) showed that both human NK cell line (NKL) and NK cells isolated from the human PBMCs express TRPV1 on both mRNA and protein level. Interestingly, authors demonstrated that protein expression of TRPV1 in the NK cell was comparable to PBMCs and U87 glioma cells, but was significantly lower from the expression level in the murine brain which confirms differences in the expression levels between different tissues. Importantly, TRPV1 expression in the human NK cells was proved to be functional as the treatment by 10 μM capsaicin-induced rise in intracellular Ca^2+^ concentration blocked by pretreatment with SB 366791 at the concentration of 10 μM or capsazepine at the concentration of 1 μM (77). The exact role of TRPV1 in the NK cells remains vague. It was shown that treatment with TRPV1 antagonist, capsazepine, caused the increase in the population of NK cells but also downregulated activation of NKT cells in the mice infected with Plasmodium (102). Nevertheless, it is hard to determine whether these effects were TRPV1-dependent.

Using flow cytometry, Assas et al. (103) demonstrated increase in the intracellular calcium concentration after capsaicin (100 μM) treatment. The murine splenic cells were gated on F480^+^, CD11c (macrophages and dendritic cells, respectively), CD19 which refers to B-cells and also on CD4^+^ and CD8^+^ standing for T helper and T cytotoxic cells. Also, it was confirmed that subpopulations of these cells express functional TRPV1 channel (103).

The data about TRPV1 expression in the B lymphocytes is very limited. To date, only studies by Soutar et al. (104) raised the issue related to TRPV1 and B lymphocytes. Authors showed that piperine-mediated inhibition of B cell proliferation, reduction of MHC II, CD40, and CD86 and decline in the production of IL-6, IL-10, and immunoglobulins. Nevertheless, piperine had the same effect on the B-cells isolated from TRPV1 KO mice and thus, the effect of piperine was TRPV1-independent. No data about TRPV1 expression in the B-cells and limited data leaves the room open for further investigation in this matter.

Unlike B cells, TRPV1 implication in T cells is a little bit more extensive, especially in the context of inflammatory and autoimmune disorders. TRPV1 was documented to be involved in the process of TCR signaling, T cell proliferation and differentiation and cytokine production (82, 97). Also, it was demonstrated that TRPV1 KO mice exhibit the reduction in the number of CD4^+^ and CD8^+^ cells in both blood and spleen when compared to wild type mice (105). The role of TRPV1 in the induction of pro- or anti-inflammatory properties of CD4^+^ cells is more elusive.

Studies by Bertin et al. (14) revealed high expression of TRPV1 in the murine splenic CD4^+^ cells on both mRNA and protein level, but also in human primary T cells and Jurkat cells. The authors also confirmed the functionality of the TRPV1 channel using patch-clamp recording along with cell stimulation with 16 μM of capsaicin. Most importantly, TRPV1 was shown to co-localize with CD4^+^ and Lck kinase and to be recruited into protein clusters upon TCR crosslinking with anti-CD3. This indicates that TRPV1 channel is involved in TCR signaling and in T cell activation. Also, TRPV1^−/−^ CD4^+^ cells exhibited significantly lower calcium influx in comparison to wild type cells when TCR was stimulated with anti-CD3 (14).

Furthermore, the role of TRPV1 in the T helper cells was linked with proinflammatory properties. Samivel et al. (106) have demonstrated that TRPV1 knockdown caused decrease in production of IL-4, IL-5, IL-6, IL-17 by murine CD4^+^ cell after TCR stimulation. Also, Jurkat cells pretreated with TRPV1 inhibitor BCTC before CD3/CD28 stimulation showed downregulation of IL-10 production. Therefore, in the model of allergic rhinitis (AR), it was shown that TRPV1 plays an important role in the activation of proinflammatory properties of Th cells. It might be used as a potential therapeutic target against inflammatory and autoimmune diseases (106). Also, Bertin et al. (107) found that the expression of TRPV1 on CD4^+^ was implicated in T cells activation and more prone differentiation into Th1 effector subtype in the murine model of colitis. Genetic or pharmacological inhibition of TRPV1 on CD4^+^ cells was also associated with the reduction of airway inflammation in allergic asthma (16).

On the other hand, Motte et al. (108) found that oral pretreatment with capsaicin in the Lewis rat experimental autoimmune neuritis caused significant reduction of sciatic nerve inflammation, the decrease in the number of infiltrating T cell and macrophages and reduction in proinflammatory cytokines production (TNFα and ING-γ) (Table 3). Interestingly, capsaicin pretreatment also caused upregulation of TRPV1 expression in the T cells and macrophages infiltrating sciatic nerve. Whether the amelioration of inflammation was TRPV1-dependent or caused by capsaicin itself, needs to be further determined. Also, studies by Acharya et al. (109) demonstrated that TRPV1 is involved in maintaining immune homeostasis in the gut via endocannabinoid system. The authors revealed that capsaicin similarly to endogenous cannabinoid anandamide [AEA] increased the frequency of CXCR1hi MΦ known for their regulatory functions. Furthermore, co-culture of naïve splenic T cells with capsaicin-pretreated myeloid cells resulted in induction of the regulatory subset of CD4^+^ producing both IL-10 and INF-γ, namely Tr1 cells via stimulation of IL-27 production by MΦ. Interestingly, capsaicin treatment does not affect both the frequency and functions of FoxP3 T reg but caused the decrease in the frequency of Th17 cells (109). The study, thus emphasizes the role of TRPV1 agonist in the induction of anti-inflammatory properties of CD4^+^ cells in the gut. However, it is worth to mention that capsaicin was shown to decrease proliferation ratio also in T cells lacking TRPV1 and thus might indicate the TRPV1-independent inhibition of colitogenic T cells (115).

**Table 3:** Effect of capsaicin and TRPV1 antagonist administration on the immune cells functioning.

**Activator/blocker**	**Dose**	**Time**	**Outcome**	**References**
Capsaicin	10 μg (per mouse)	24 h	↑ frequency of CXCR1hi MΦ ↑ induction of CD4^+^ ↑ production of IL-10 and INF-γ ↓ frequency of Th17	(109)
	10–25 μg (orally administered)	4 days	↑ upregulation of anti-inflammatory cytokines such as IL-10 by macrophages ↓ autoreactive T cell proliferation	(96)
	12–100 μM	360 s	↑ dose dependent increase of calcium levels in splenic DCs ↑ release of CGRP	(83)
	100 μM	360 s	↑ intracellular Ca^2+^ concentration in DC, B-cells, T helper, and T cytotoxic cells	(103)
	5–100 μM	16 h	↑ dose-dependent effect on DCs maturation and migration to lymph nodes	(97)
	16 μM 1 and 10 μM	<20 s	↑ inward current in CD4^+^ T cells on patch-clamp recordings, ↑ intracellular Ca^2+^ level based on Fura-2 calcium assay ↑ proinflammatory properties of CD4^+^	(14)
	-	-	No alterations in calcium influx or cation currents in neutrophils	(99)
	10-100 μM	2 h	↓ degranulation, cytotoxicity, cytokine production (IFN-γ, TNF-α) by NK cells	(77)
	10 μM	180 s	↑ intracellular Ca^2+^ level in neutrophils from human patients	(101)
	0.01–10 mg/kg daily by oral gavage	10 days, 16 days,	↓ sciatic nerve inflammation, ↓ number of infiltrating T cells and macrophages, ↓ proinflammatory cytokines production (TNFα and ING-γ) in a Lewis rat experimental autoimmune neuritis model	(108)
	1 μM	5–10 s (membrane current) 60 s (intracellular Ca^2+^ level)	↑ intracellular Ca^2+^ levels in sensory neurons and TRPV1-expressing HEK293 cells no changes in the intracellular calcium concentration or membrane current in the DCs	(98)
	10 μM	24 h	↓ production of pro-inflammatory cytokines in primary murine macrophages (MCP-1 and IL-6)	(95)
Capsazepine	-	10 min before capsaicin treatment	↓ DCs maturation	(97)
	50 μg/animal	Injections twice a day for 6 days	↑ population of NK cells ↓ activation of NKT cells	(102)
	1 μM	30 min before Capsaicin treatment	↓ intracellular Ca^2+^ concentration in NK cells	(77)
	0.1 mM	30 min	↓ intracellular Ca^2+^ level in neutrophils from human patients	(101)
	10 μM	30 min	↓ pro-inflammatory cytokines production IL-6, IL-1β, and IL-18 ↓ COX-2 expression in the LPS-stimulated murine macrophages	(41)
	10 μM	1 h	↑ pro-inflammatory effect	(95)
BCTC	0.1 μg/ml; 1 μg/ml; 10 μg/ml	1 h	↓ secretion of cytokines (IL-4, IL-5, IL-6, IL-10, IL-17, and IFN-γ) murine CD4^+^	(106)
TRPV1 genetic ablation	-	-		
Evodiamine	0.5 μM	24 h	↓ production of pro-inflammatory cytokines (MCP-1 and IL-6) by macrophages	(95)
SB366791	10 μM	30 min before capsaicin treatment	↓ intracellular Ca^2+^ concentration in NK cells	(77)
SLS	50 mL of bacterial supernatant	30 min	↑ CGRP release ↓ neutrophils defense against *Streptococcus pyogenes* ↑ bacterial infection	(110)
XEN D0501	10 mg/kg	1 h before and 30 min after OVA challenge	↓ airway inflammation ↓ IgE levels ↓ lung function changes ^*^ targeting TRPV1 on CD4^+^ cells	(16)
Cutaneous light stimulation	-	-	↑ production of IL-23 by dermal DCs ↑ production of IL-17A by γδ T ↑ host defense against pathogens in TRPV1-Ai32 optogenetic mouse model	(111)
TRPV1^+^ neurons ablation by RTX	Increasing doses: 30, 70, and 100 mg/kg	Applied subcutaneously on 3 consecutive days	↓ IL-23 production ↓γδ T cells number ↑ in fungal burden	(112)
	Increasing doses: 30, 70, and 100 mg/kg	Applied subcutaneously on 3 consecutive days	↓ IL-23 production ↓ recruitment of proinflammatory cells	(113)
TRPV1 genetic ablation			↑ animals survival rate ↑ cytokine induction ↑ lung bacterial clearance	(114)

An important aspect to consider is the capsaicin dose used in the studies. It is widely known that capsaicin triggers calcium influx into nerve cells or transfected with TRPV1 HEK293 cells at relatively low doses (1 μM or less). The same effect was not observed, however, in most of the studies on the immune cells at the same doses. The use of higher doses of capsaicin raises the question about receptor-dependent signaling. In contrast to neurons, TRPV1 expression is lower in other cell types such as immune cells or keratinocytes (31, 66). Correlation between TRPV1 expression level and receptor-dependent calcium influx caused by capsaicin in other cell types, including immune cells, should be carefully addressed in further studies.

Another important aspect that should not be overlooked is an indirect impact of TRPV1 activation on the immune cell functioning. Currently, it is known that nociceptors with high expression of TRPV1 channel and immune cells act together especially at lymphoid tissues (lymph nodes) and barrier tissues such as skin, mucosa or gut (111, 116, 117). Activation of TRPV1^+^ neurons leads to the release of neuropeptides such as CGRP which affects the cytokine production, recruitment or polarization of the immune cells (118).

Cohen et al. (111) found that cutaneous TRPV1^+^ neurons are located close to dermal DCs. More importantly, the authors revealed that activation of neuronal TRPV1 channel was associated with the production of IL-23 by dermal DCs and further production of IL-17A by γδ T cells that elicit host defense against pathogens such as *Candida albicans* and *Staphylococcus aureus*. Similarly, Kashem et al. (112) demonstrated that the ablation of TRPV1^+^ sensory neurons led to a decrease in IL-23 production, γδ T cells number and subsequent increase in fungal burden. Nevertheless, several studies reported that the ablation of TRPV1^+^ neurons might be beneficial in the treatment of inflammatory disorders and some infection. Riol–Blanco et al. (113) found that the pharmacological or genetic ablation of TRPV1^+^ fibers is followed by a decrease in IL-23 production and recruitment of inflammatory cells in the murine model of psoriasiform skin inflammation. The authors confirmed that TRPV1^+^ neurons are a crucial element of the IL-23/IL-17 pathway and thus, might be used as a target to modulate the local immune response. Pinho–Riberio et al. (110) demonstrated that the induction of severe hyperalgesia, associated with infection with *Streptococcus pyogenes*, was mediated by direct activation of nociceptors by bacterial toxin SLS. Activation of TRPV^+^ neurons by SLS results in CGRP release which in turn suppresses the neutrophils defense against the pathogen. Authors, also found that silencing the nociceptors by botulinum neurotoxin A or use of CGPR antagonist stopped the progression of infection. Also, Baral et al. (114) found that activation of TRPV1^+^ nociceptors is associated with suppression of the host defense in the course of *Staphylococcus aureus* pneumonia. The authors showed that the ablation of TRPV1^+^ nociceptors increased survival rate and decreased bacterial burden in a murine pneumonia model.

The neuro-immune crosstalk in many aspects remains elusive. Nevertheless, communication between the nervous and immune system is a promising field for future research and is of great importance for medical purposes.

## Conclusions and Perspectives

TRPV1 ion channel is a polymodal cellular receptor that can perceive different stimuli, integrate them and translate to the language of calcium-based signals. Therefore, it constitutes an important link between the extracellular environment and cellular response. Recent studies are increasingly turning attention to TRPV1 as a potential target to treat different disorders including inflammation, autoimmune diseases, and cancer. There are no doubts that TRPV1-based signaling might take part in the regulation of cellular functions in both health and disease. However, TRPV1 display a promiscuous nature with regard to its expression and activation. In many cases, the results regarding its role in inflammation, cancer, and immunity are contradictory. The experimental design, as well as the results analysis, requires thus greater cautions. It is important to take into consideration differences that result from study type (*in vitro* vs. *in vivo*), cell type and subsequent expression level, possible splice variants, agonist dose and time interactions, synergistic or antagonistic influence of endogenous agonists, or point mutations that can modulate channel activity. More efforts need to be put to distinguish the effect of TRPV1 activation from capsaicin action, especially with particular emphasizes on doses and treatment duration in both *in vitro* and *in vivo* studies. Furthermore, the dynamics of TRPV1 expression has to be more precisely determined along with the phenomenon called desensitization and internalization which might influence the final outcome. Moreover, the exact molecular pathways that TRPV1 is involved in are not well-understood and thus special precautions need to be taken. Lastly, more specific activators and blockers of TRPV1 ion channel should be used in order to assess its function more precisely and avoid off-target effects.

Studies on the role of TRPV1 channel are complex and demanding, however, they also offer an attractive treatment possibility for several diseases by modulation of cell functions. Deepening the knowledge about TRPV1 functioning contribute toward better understanding of cellular processes important not only in the inflammation or malignant transformation. Recent attempts showed that TRPV1 can be used in the remote regulation of gene expression and modulation of insulin secretion (119). Also, TRPV1 KO mice exhibit youth-metabolism and are long living which implicates TRPV1 in studies related to obesity, metabolic dysfunctions, and aging. Therefore, further studies addressing the role of TRPV1 might be beneficial also for other fields of biomedical sciences.

## Author Contributions

JB and DK: manuscript writing. JB, DK, and IS: table and figures preparation. PB and KM: critical review, help with conception, and expertise.

### Conflict of Interest

The authors declare that the research was conducted in the absence of any commercial or financial relationships that could be construed as a potential conflict of interest.
